# West London Hospital: The Late Duke of Abercorn

**Published:** 1913-06-14

**Authors:** Arthur Hill, Geo. F. Marshall

**Affiliations:** Chairman, "Abercorn Memorial" Executive Committee.; Chairman, West London Hospital.


*To the Editor of* The Hospital.

### West London Hospital: The late Duke of Abercorn.

*To the Editor of* The Hospital.

Sir,—With the concurrence of His Royal Highness Prince Arthur of Connaught, president of the hospital, we ask you to be good enough to allow space in your influential paper to bring under the notice of your readers the following particulars concerning an effort which is now being made in memory of the late Duke of Abercorn, who, as the former president and chairman of the hospital, was throughout a lengthened period closely associated with its daily life and work. It is generally felt that no more fitting tribute can be paid to his memory than by erecting new accommodation for the nursing staff and naming it the "Abercorn Home for Nurses." The matter was submitted to His Royal Highness Prince Arthur of Connaught on the occasion of his visit on May 21, and he then authorised us to state that it met with his entire approval. At present the nurses are crowded into several private houses in various parts of the hospital premises. Owing to want of space it is impossible to make adequate provision for our nurses, and most of the bedrooms are shared by two or three. The question of the proper housing of the nursing staff was one in which the late Duke took the greatest personal interest, and in the hope of improving matters an appeal was made under his direction, which resulted in some £2,000 being subscribed towards a new "Home." A further £10,000 is required to carry out the work, and it is earnestly hoped that sufficient help will be forthcoming to enable the late President's wish to be realised, and at the same time afford an opportunity for the board to associate his name for ever with a work which he had so much at heart. Contributions, however small, will be very gratefully received, and may be addressed to us at the hospital.—Yours faithfully,

Arthur Hill,
Chairman, "Abercorn Memorial" Executive Committee.
Geo. F. Marshall,
Chairman, West London Hospital.

P.S.—Up to the present a total sum of £3,200 has been received.

### Verification of Death.

*To the Editor of* The Hospital.

